# AI and Blockchain-Based Secure Data Dissemination Architecture for IoT-Enabled Critical Infrastructure

**DOI:** 10.3390/s23218928

**Published:** 2023-11-02

**Authors:** Tejal Rathod, Nilesh Kumar Jadav, Sudeep Tanwar, Zdzislaw Polkowski, Nagendar Yamsani, Ravi Sharma, Fayez Alqahtani, Amr Gafar

**Affiliations:** 1 Department of Computer Science and Engineering, Institute of Technology, Nirma University, Ahmedabad 382481, India; 20ftphde39@nirmauni.ac.in (T.R.); 21ftphde53@nirmauni.ac.in (N.K.J.); 2 Department of Humanities and Social Sciences, The Karkonosze University of Applied Sciences, 58-506 Jelenia Galora, Poland; zdzislaw.polkowski@kans.pl; 3 Department of Computer Science and Artificial Intelligence, SR University, Warangal 506371, India; nagendar.y@sru.edu.in; 4 Centre for Inter-Disciplinary Research and Innovation, University of Petroleum and Energy Studies, P.O. Bidholi Via-Prem Nagar, Dehradun 248007, India; ravisharmacidri@gmail.com; 5 Software Engineering Department, College of Computer and Information Sciences, King Saud University, Riyadh 11437, Saudi Arabia; fhalqahtani@ksu.edu.sa; 6 Mathematics and Computer Science Department, Faculty of Science, Menofia University, Shebin Elkom 6131567, Egypt; atolba@science.menofia.edu.eg

**Keywords:** Internet of Things, critical infrastructure, artificial intelligence, security, data poisoning attacks, blockchain network

## Abstract

The Internet of Things (IoT) is the most abundant technology in the fields of manufacturing, automation, transportation, robotics, and agriculture, utilizing the IoT’s sensors-sensing capability. It plays a vital role in digital transformation and smart revolutions in critical infrastructure environments. However, handling heterogeneous data from different IoT devices is challenging from the perspective of security and privacy issues. The attacker targets the sensor communication between two IoT devices to jeopardize the regular operations of IoT-based critical infrastructure. In this paper, we propose an artificial intelligence (AI) and blockchain-driven secure data dissemination architecture to deal with critical infrastructure security and privacy issues. First, we reduced dimensionality using principal component analysis (PCA) and explainable AI (XAI) approaches. Furthermore, we applied different AI classifiers such as random forest (RF), decision tree (DT), support vector machine (SVM), perceptron, and Gaussian Naive Bayes (GaussianNB) that classify the data, i.e., malicious or non-malicious. Furthermore, we employ an interplanetary file system (IPFS)-driven blockchain network that offers security to the non-malicious data. In addition, to strengthen the security of AI classifiers, we analyze data poisoning attacks on the dataset that manipulate sensitive data and mislead the classifier, resulting in inaccurate results from the classifiers. To overcome this issue, we provide an anomaly detection approach that identifies malicious instances and removes the poisoned data from the dataset. The proposed architecture is evaluated using performance evaluation metrics such as accuracy, precision, recall, F1 score, and receiver operating characteristic curve (ROC curve). The findings show that the RF classifier transcends other AI classifiers in terms of accuracy, i.e., 98.46%.

## 1. Introduction

The Internet of Things (IoT) is a prominent technology that plays an essential role in futuristic wireless communication and IoT-based applications, such as smart industry, smart city, 6G-based smart network communication, and smart healthcare systems. IoT comprises different smart objects (sensors) that work together to collect real-time data from various physical devices for a future-proof growth path. Nowadays, many industries, such as healthcare, manufacturing, automation, and agriculture, adopt IoT-based solution technology to improve their existing systems and fulfill future technological demands. There is a significant consideration of IoT devices in critical infrastructures, such as water treatment plants, nuclear power plants, smart grids, thermal plants, etc., that enhance the performance of the relevant system in terms of quality standards, volume manufacturing, interoperable and scalable [1]. The sensors attached to the critical infrastructure exchange data with each other for a collaborative operation, such as controlling chlorine levels in the water treatment plant, managing pressure and temperature in nuclear power plants, etc. They utilize different messaging and communication protocols, such as message queue telemetry transport (MQTT), advanced message queuing protocol (AMQP), and the International Electrotechnical Commission’s (IEC) standard protocols for data exchange between IoT-enabled critical infrastructure [2].

IoT devices provide significant value to the oil, gas, telecom, and electricity generation industries, which in turn boost the nation’s economy [3]. Each physical device in the critical infrastructure environment poses sensitive data to operate different core systems and business operations (e.g., online transactions). However, the precarious link on which most of the critical infrastructure communicates is lured by the attackers, who exploit the data exchange between different devices to propagate their attack surface [4]. In addition, improper credential configurations and unpatched vulnerabilities make critical infrastructure vulnerable to system-level attacks (e.g., privilege escalation attacks). Here, the attacker first performs reconnaissance of different IoT-based devices and their respective network interfaces to discover open vulnerabilities; then, it exploits the vulnerabilities and performs attacks, such as data manipulation, distributed denial-of-service (DoS), session hijacking, and malware, to manipulate the behavior of critical infrastructures. To overcome these issues, many researchers have proposed security-based solutions such as incident response, threat intelligence [5], artificial intelligence (AI) [6], blockchain [7], etc. They perform vulnerability assessments that identify the unpatched devices and introduce multi-factor authentication approaches to enhance network security and reduce the attack surface.

For example, Liu et al. [8] reviewed smart world critical infrastructures and cyber-physical systems such as smart transportation, smart grid, smart industries, and smart manufacturing. They investigated threats and vulnerabilities that raise security and privacy issues in IoT-based critical infrastructures. Moreover, they identify the attacks that damage critical systems. Furthermore, they carried out a case study highlighting the risk of cyberattacks with their defensive mechanisms in smart transformation. In article [9], the authors addressed security threats in IoT-based smart grid applications. They investigated big data attacks that bypassed bad data detection and proposed a replay scheme based on direct or alternating current state estimation. Furthermore, Namasudra et al. [10] proposed a deoxyribonucleic acid (DNA) cryptography and steganography approach to secure the confidential data of the critical infrastructure stored on the cloud server. They discussed the prevention mechanisms of diverse cyber attacks, such as DDoS, collision, ciphertext-only, man-in-the-middle (MITM), and statistical attacks. Furthermore, Miguelez et al. [11] proposed Industry 4.0-based IoT solutions in urban areas to enhance water supply and purification in smart cities. They introduced an IoT-based architecture for managing the wastewater treatment process, monitoring water levels, controlling chemical processes, and ensuring efficient water supply. Moreover, their suggested architecture provides insight into the functionality of this critical infrastructure within the context of Industry 4.0.

Recently, edge computing, cloud computing, artificial intelligence (AI), and blockchain technology have attracted a lot of attention by ensuring security and privacy in IoT-based critical infrastructures [12]. Based on this background, Hayyolalam et al. [13] introduced edge computing and AI-based approaches for the cloud and IoT-based healthcare systems. Here, edge computing decreases energy consumption and latency, while AI predicts and detects high-risk diseases and provides efficient treatment with the minimum medical expense. They identified the existing schemes’ limitations and proposed a deep reinforcement-learning smart healthcare model. Furthermore, Liu et al. [14] proposed a blockchain-driven electronic healthcare system that facilitates the security and privacy of the patient’s medical record. Furthermore, Otoum et al. [15] presented blockchain with federated learning. In their proposed scheme, blockchain offers security, and a federated learning-based framework fulfills network trustworthiness by facilitating decentralization.

The aforementioned works show that much research has been done in the field of IoT-based critical infrastructures for device security and user privacy. Nevertheless, only a few have focused on the security of data dissemination between IoT-enabled devices. Furthermore, many researchers have adopted AI algorithms to predict attacks in the IoT-driven critical infrastructure intelligently; however, they have yet to investigate an approach where their AI models are corrupted using data poisoning attacks by attackers. Researchers have also used blockchain-enabled solutions for critical infrastructure. Nevertheless, they have not discussed their solutions for the detection or prevention of data poisoning attacks. Motivated by this, we propose an AI and blockchain-driven intelligence and secure data dissemination architecture for IoT-based critical infrastructure. We applied different AI classifiers, such as random forest (RF), decision tree (DT), support vector machine (SVM), perceptron, and Gaussian Naive Bayes (GaussianNB), to a standard dataset to bifurcate malicious and non-malicious IoT data. To enhance the performance of the proposed architecture and overcome the overfitting issue, we introduced feature selection approaches such as principal component analysis (PCA) and Explainable AI (XAI) that perform dimensionality reduction before the classification in the data. Furthermore, AI models are secured from data poisoning attacks by analyzing and removing anomalous data from the dataset. The performance of the proposed architecture is evaluated using different performance evaluation parameters, such as accuracy, precision, recall, and F1 score. Subsequently, we introduced an interplanetary file system (IPFS)-based blockchain designed to reduce mining costs by utilizing event logs while providing secure storage for critical infrastructure data.

### 1.1. Motivation

The motivation for the secure data dissemination architecture in IoT-based critical infrastructure can be defined as follows:

- Nowadays, IoT sensors are connected to critical infrastructures to exchange sensitive data between devices. However, IoT devices use legacy infrastructure and weak protocols that are vulnerable to different cyberattacks, such as botnets, DoS attacks, ransomware, malicious node injection, and data poisoning attacks.
- Most of the researchers in this domain use AI-enabled solutions for IoT-based critical infrastructure [5,10,13]. Their proposed AI solutions for security threat detection in IoT-based critical infrastructure are not resistant to data poisoning attacks, i.e., the dataset is itself corrupted or has been tampered with by the attackers. Moreover, most of the existing works have lower accuracy in classifying attack and non-attack data for critical infrastructure. Furthermore, they have not used PCA and XAI approaches to include essential features that can maximize the performance of AI models.
- In addition, the researchers who adopted blockchain-based solutions are computationally expensive because they have to process both attack and non-attack data [14,15]. They have not used any intelligence or filtering mechanism that can bifurcate attack and non-attack data, thus reducing the computation overhead of the blockchain.
- Based on the aforementioned facts, there is a requirement for an amalgamation of AI and blockchain for security enhancements in IoT-enabled critical infrastructure. Therefore, we proposed an AI and blockchain-based intelligent and secure data dissemination architecture for IoT-based critical infrastructure.

### 1.2. Research Contributions

The following are the major research contributions to this paper:

- To propose an AI and blockchain-driven secure data dissemination architecture for anomaly detection in IoT-enabled critical infrastructure.
- We applied diverse future selection methods, such as PCA and XAI, that fetch the important future from the dataset to reduce the computation overhead. Furthermore, we employed different AI classifiers, such as RF, DT, SVM, GaussianNB, and perception, that classify the malicious and non-malicious data by training the AI classifier on the standard dataset comprising network traffic and communication protocol between IoT sensors for the critical infrastructure. Furthermore, the AI models are secured against data poisoning attacks using an isolation forest algorithm that deteriorates the performance of AI training.
- Moreover, we incorporated an IPFS-driven blockchain network that ensures the secure data storage of the IoT-enabled critical infrastructure’s data.
- The performance of the proposed architecture is evaluated by considering distinct performance parameters, such as accuracy, precision, recall, F1 score, receiver operating characteristic (ROC) curve, and scalability.

### 1.3. Organization

The entire paper is organized into five sections. Section 2 discusses the related work. In Section 3, we present the system model and problem formulation. Furthermore, in Section 4, we present our proposed architecture. Results and discussion of the proposed architecture are discussed in Section 5. Finally, Section 6 concludes the paper.

## 2. Related Work

A lot of research work has been carried out to secure data from the IoT-based critical infrastructure. Across the globe, the research community uses various AI-based approaches for different critical infrastructures such as thermal plants, smart grids, smart healthcare, nuclear power plants, water treatment plants, etc. For example, Mosavi et al. [16] perform real-time transient stability assessment in power systems. For that, they proposed a convolutional neural network (CNN)-based framework known as twin convolutional SVM. They compare the performance of the adopted scheme with the SVM recursive time warp kernel SVM-radial basis functions (Euclidean distance). The results show that the proposed scheme achieves higher accuracy than the aforementioned kernel-based schemes. Furthermore, in [17], Chang et al. addressed cybersecurity issues in industrial control systems (ICS). They proposed an anomaly detection approach combining two semi-supervised algorithms, k-means clustering and convolutional autoencoders. Moreover, they presented the k-means normal behavior model and cluster model for each attribute of the gas pipeline and water storage tank dataset. They also performed feature selection on the dataset that helped enhance the performance of the proposed scheme. They compared the adopted scheme with the baseline approaches. Their findings show that the proposed scheme achieves good performance for the gas pipeline dataset in terms of a 4% and 21% higher F1 score compared with the Feng et al. [18] and k-means methods, respectively. In contrast, for the water tank dataset, the proposed scheme performs worse than a hidden Markov model-based method [19] in terms of accuracy, precision, and recall.

Alhaidari et al. [20] tackled security issues by controlling denial of service (DDoS) attacks on the supervisory control and data acquisition systems. To identify the attack pattern, they proposed different machine learning (ML) algorithms such as NB, RF, and J48 [21,22]. They considered the KDDCup’99 dataset, which contains different types of attacks. Their results show that the RF algorithm performs better than the NB and J48 algorithms in terms of accuracy. Furthermore, Elnour et al. [23] have combined isolation forest and CNN for cyberattack prevention in the ICS. Therefore, they proposed a hybrid attack detection scheme to secure water treatment plants. They used a one-dimensional CNN for the feature extraction and an isolation forest approach for the attack detection. Their adopted scheme detects 70% of attacks in the dataset. It also increases the overall accuracy and reduces computational complexity.

In the article [24], Rakesh et al. proposed a water quality monitoring approach using different ML techniques such as NB, RF, and LR in the IoT-based water treatment plant. They compared the theoretical results with the experimental results. Their finding shows that the proposed approach gives good water quality observation system results. Furthermore, Puthal et al. [25] worked on fake data identification in the IoT-enabled critical infrastructure environment. They combined IoT networks with edge data centers and proposed a DT-based DecisionTSec approach that offers security in the communication channel. The simulation results show the proposed security approach resists the brute force and replay attacks. Moreover, it does not affect the overall throughput of the system.

Recently, in [26], the author proposed an automatic behavioral abstraction technique based on the neural network for anomaly detection in smart cyber-physical systems. They worked on the SWaT dataset, which is based on the sewage water treatment plant. They are able to detect 88 % attacks in the SWaT dataset with a 1% false positive rate. Furthermore, Sharmeen et al. [27] introduced semi-supervised and deep learning approaches to prevent cyberattacks on smart water treatment and distribution networks. They used RF, extra tree, DT, SVM, and K-means algorithms for the supervisory control and data acquisition system-based water treatment plant. Their findings show that the extra tree classifier gives better results compared with the other approaches in terms of accuracy, precision, recall, and F1 score. In the above work, the authors have applied diverse AI-based algorithms to worked with all the IoT-driven critical infrastructure. Some of them have concentrated on anomaly detection, while the majority have not applied dimensionality reduction through PCA and feature extraction using XAI. In their proposed work, they worked with the entire future of the dataset, which increased the computational cost. Furthermore, the authors have not used blockchain-based security with AI-based solutions. Hence, we explored literature where the research community amalgamates blockchain technology with AI algorithms. 

For example, Khan et al. [28] proposed a resource-efficient solution for IoT applications. They used ANN, SVM, DT, and a deep extreme learning machine approach with blockchain. Furthermore, Gu et al. [29] presented ML and blockchain-driven schemes for quality control in manufacturing. They used KNN, XGboost, ANN, and XGB max algorithms. They also considered different security hurdles and attacks, such as denial-of-service (DoS) attacks, man-in-the-middle attacks, distributed DoS, and brute force attacks. In the aforementioned work, the authors tackled security issues in the IoT-based critical infrastructure. They have applied AI and blockchain-driven approaches to the various IoT-based critical infrastructures. However, many of them have not applied feature-section approaches such as PCA and XAI that enhance the performance of the AI classifier. These solutions work with the conventional blockchain, where the data is stored in the blockchain network. However, they have not considered IPFS-enabled blockchain networks that use event logs to securely store data in blockchain networks by minimizing mining costs. Furthermore, they have not considered data poisoning attacks in anomaly detection [30]. Thus, we integrate AI with IPFS-based blockchain for IoT-driven critical infrastructures that facilitate data poisoning attack detection with secure, efficient, and reliable storage. Table 1 shows the comparative analysis of existing approaches for anomaly detection in IoT-based critical infrastructure.

## 3. System Model and Problem Formulation

**Figure 1:**
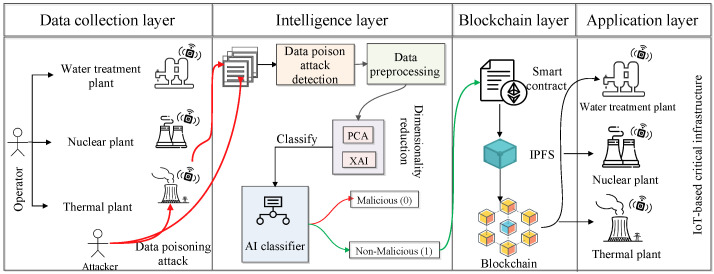
Proposed architecture.

In this section, Figure 1 shows the system model for the proposed architecture. Here, we have considered IoT-based critical infrastructures such as water treatment plants, nuclear power plants, thermal plants, etc., as $c_{T}$. The application users, known as operators $O\in O_{1},O_{2},\ldots ,O_{n}$, operate these applications and take readings from the different sensors. Meanwhile, one of the people from that operator intended malicious activity on the critical infrastructure and attacked the sensor of the critical infrastructure. As a result, the operator who is taking the readings $R\in R_{1},R_{2},\ldots ,R_{n}$ from the sensors gets the tempered data and stores that data in the dataset *D*. The above-mentioned entity can be represented as follows:

(1) $$\begin{array}{c} O_{i}\overset{\mathrm{operates}}{\to }C_{T} \end{array}$$

(2) $$\begin{array}{c} O_{i}\overset{\mathrm{reading}}{\to }R_{i}\in C_{T} \end{array}$$

(3) $$\begin{array}{c} \forall i=1,2,\ldots ,n \end{array}$$

(4) $$\begin{array}{c} O{}^{\prime }\overset{\mathrm{attacks}}{\to }D_{C_{T}},O{}^{\prime }\neq O \end{array}$$

To collect the data from the sensor, the operator considered different features with different timestamps and stored that data in the comma-separated values (CSV) file. Furthermore, we applied AI classifiers that classify malicious and non-malicious data for IoT-enabled critical infrastructure. For this process, we considered the standard dataset, the IEC 60870-5-104 intrusion detection dataset, which has different features with class labels 0 and 1. Here, class 0 means malicious data, and 1 means non-malicious data.

The proposed architecture has a data collection layer, where the operator collects data from the sensor and stores that data in CSV files. Furthermore, the dataset is passed to the intelligence layer, where pre-processing, dimensionality reduction, data splitting, and validation are performed. In the critical infrastructure scenario, the data is required to be secure. However, the data is affected by the data poisoning attack. Therefore, we proposed a secure data dissemination architecture for IoT-based critical infrastructure. The mathematical description of the secure data dissemination problem is given as follows.

(5) $$\begin{array}{c} O=\sum_{i=0}^{n}\max\left(R\right) \end{array}$$

In this paper, our objective is to maximize the security of the data for the IoT-based critical infrastructure.

## 4. The Proposed Architecture

This section shows the workings of the proposed architecture, which is an AI and blockchain-driven intelligent and secure data dissemination architecture for IoT-enabled critical infrastructure. We proposed different AI classifiers that maximize the classification of whether the data is malicious or non-malicious. For that, we considered RF, DT, SVM, perceptron, and LR. The proposed architecture has a data collection, intelligence, blockchain, and application layer. The detailed discussion of each layer is as follows.

### 4.1. Data Collection Layer

In this layer, we have various IoT-based critical infrastructures $C_{T}$, such as water treatment plants, thermal plants, nuclear power plants, etc. Different types of IoT sensors are attached to these critical infrastructures. The operator *O* takes reading *R* from that sensor and stores the data into CSV files. However, among these operators, one person (an attacker) intentionally manipulates the sensor. As a result, the operator who is taking the result from the sensor of critical infrastructure gets tempered data, and that data is stored in the CSV file. This type of data manipulation activity is known as a poisoning attack. This attack affects the accuracy of the AI classifier. We applied a data poisoning attack detection approach in the sub-sequence layer, which is the intelligence layer. For that, we forward the collected data file to the intelligence layer that performs further processing, avoids data poisoning attacks, and classifies the data into malicious and non-malicious categories.

### 4.2. Intelligence Layer

The intelligence layer comprises dataset description, data poisoning attack prevention, data pre-processing, and AI-based classification.

#### 4.2.1. Dataset Description

We have collected the IEC 60870-5-104 intrusion detection dataset from the IEEE dataport [32]. In this dataset, IEC 60870-5-104 is an industrial protocol that is used for IoT-based critical infrastructures. The dataset contains various network configurations and traffic features, such as packet size, bandwidth, etc. The dataset includes 83 different features with numerical values.

(6) $$\begin{array}{c} Cl_{1},Cl_{2},\ldots ,Cl_{n}\in D_{T},n=1,2,\ldots ,83 \end{array}$$

#### 4.2.2. Data Poisoning Attack Prevention: Isolation Forest

The data poisoning attack refers to the technique in which the malicious user tampers the training datasets by introducing malicious data. The malicious user performed malicious activity on the sensor, manipulated the information, and produced inaccurate outcomes. The dataset has data poisoning attacks, such as human error, injection attacks, etc., that degrade the performance of the IoT-based critical infrastructure. To prevent the data poisoning attack on the dataset, we proposed an ensemble learning approach known as isolation forest that detects and removes the outlier. To accomplish this, we leveraged the Numpy, Pandas, Matplotlib, and Sklearn libraries within a Google Colab notebook. It calculates the anomaly score as follows.

(7) $$\begin{array}{c} A\left(n,q\right)=2^{-\frac{E(r(n))}{s(q)}} \end{array}$$

where *n* is the data point for which we calculate the anomaly score. Furthermore, *q* is the total number of data points in the dataset. r(n) and E(r(n)) are the path length and expected path length of the data point *n*, respectively. Furthermore, s(q) represents the normalization factor, which corrects the expected path length for the given number of data points. The aforementioned equation gives an anomaly score between 0 and 1.

#### 4.2.3. Data Pre-Processing

Before passing the dataset to the AI classifier, we apply data pre-processing to eliminate missing values, infinity values, and noise from the dataset. Moreover, we use PCA and XAI feature selection techniques for dimensionality reduction in the dataset [33,34]. For the pre-processing of the dataset, we identify the missing values in the dataset. There are three imputation techniques to replace the missing values, such as mean, median, and mode. From these three, we applied the mean imputation technique to replace the missing values in the dataset. To calculate the mean, we have to take the average value of a series of numbers from the particular feature. Mathematically, the mean is calculated as follows.

(8) $$\begin{array}{c} \bar{\chi }=\frac{\sum_{i=0}^{n}\chi }{N} \end{array}$$

where $\bar{\chi }$ is the mean of the particular feature (column), $\sum_{i=0}^{n}\chi$ is the summation of all the values in that column, and N is the total number of elements (rows). Furthermore, we get infinity and NaN values from the dataset. We replaced those values with zero. Furthermore, we used MinMaxScaler from the Sklearn library in the Google Colab notebook. Furthermore, we proposed a Z-score normalization approach to normalize the dataset. The mathematical representation of the Z-score is as follows.

(9) $$\begin{array}{c} Z=\frac{\chi_{\upsilon }-M\left(\chi_{\upsilon }\right)}{S_{\upsilon }} \end{array}$$

where the number of standard deviations $S_{\upsilon }$ is away from the mean value $M(\chi_{\upsilon })$. While forming the classification problem, we observed that the dataset has a large number of features that raise the issue of overfitting. To mitigate this issue, we proposed dimensionality reduction techniques such as PCA and XAI, which are also known as feature selection approaches. Dimensionality reduction techniques remove the least important features from the dataset.

**Figure 2:**
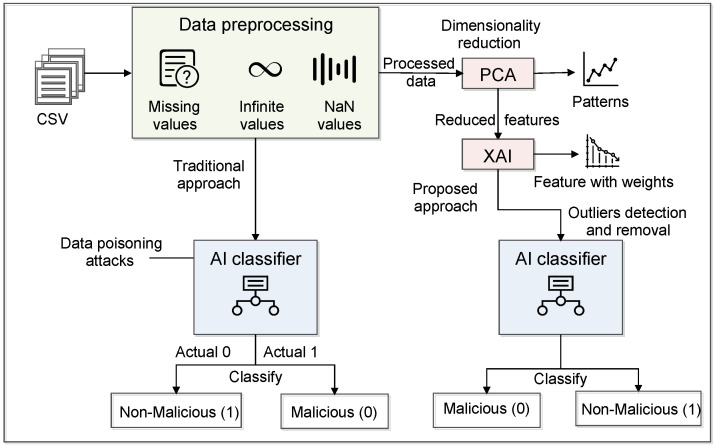
A detailed representation of Intelligence layer.

Figure 2 shows the workings of the traditional and proposed approaches. Initially, we fed the data into AI classifiers without applying any feature selection or dimensionality. The proposed architecture follows the steps of pre-processing, feature selection, and classification that give better performance to deal with the data poisoning attack in the IoT-based critical infrastructure. The first feature selection approach is PCA, where we have reduced the number of features by applying five steps [35]. The first step is standardization, where we have to standardize the range of the continuous initial variables so that each one of them contributes equally to the analysis. This method is also known as normalization, where we transform the data to comparable scales that can prevent biased results. The next step is covariance matrix computation, where we identify the correlation between input data. Furthermore, it calculates the eigenvectors and eigenvalues from the covariance matrix. After that, sort the eigenvalues in decreasing order. Here, sorting is performed in such a way that there is no loss of information about which eigenvalue corresponds to which eigenvector. The last step of PCA is to form a new feature set in terms of the matrix. In this matrix, we kept column entries as eigenvectors, and every row of the eigenvector matrix corresponds to one eigenvector value. In the end, we transpose the selected eigenvector as column entries, which is called a feature matrix. These techniques use the PCA class from the decomposition module of the Sklearn library in the Google Colab notebook.

Initially, we have 83 features in the dataset. Once we apply the PCA approach, we get 35 important features from the dataset. This feature count is also large enough to pass into the classifier. Therefore, we used the XAI approach, which further reduces the feature size and gives important features their weights (scores). For XAI, we apply the Explain like I am a 5 (ELi5) ML framework that returns the weights of each feature. It uses different libraries, such as Sklearn, Keras, XGBoost, and LightGBM. Furthermore, we get 20 important features that have a higher weight. Once we finalize our dataset, we pass it to the different AI classifiers such as RF, DT, SVM, perceptron, and GaussianNB.

#### 4.2.4. AI-Based Classification

Here, the dataset is split into two parts, training and testing, which have an 80:20 ratio. We used the train_test_split class from the model_selection module of the Sklearn library. Furthermore, the training dataset is fed into the AI classifier (α), which classifies the data into binary classes as class 0 and class 1. Here, class 0 means malicious data (Π), and class 1 means non-malicious data (σ) for the IoT-based critical infrastructure. Furthermore, we use the test dataset β that validates the result based on the training.

(10) $$\begin{array}{c} \alpha \overset{\mathrm{fit}}{\to }\varphi \overset{\mathrm{predict}}{\to }class\left\{\begin{array}{cc} \Pi & \left(0\right)\\ \sigma & \left(1\right) \end{array}\right. \end{array}$$

(11) $$\begin{array}{c} \beta \overset{\mathrm{fit}}{\to }\varphi \overset{\mathrm{predict}}{\to }class\left\{\begin{array}{cc} \Pi & \left(0\right)\\ \sigma & \left(1\right) \end{array}\right. \end{array}$$

where φ is the different AI classifiers, such as RF, DT, SVM, perceptron, and GaussianNB. In our Google Colab notebook, we used the RandomForestClassifier and GradientBoostingClassifier classes from the ensemble module of the Sklearn. Similarly, we import the SVC class from the SVM module, Perceptron from $linear_{m}odel$, and GaussianNB from the $naive_{b}ayes$ module of the Sklearn library. After applying all the classifiers, we get the best results from the RF classifier. In RF classification, each tree’s prediction is a class label. The final RF prediction will take a majority vote over these predictions that work well for classification. It also reduces the overfitting issue in DT and minimizes the variance, which improves the accuracy. Finally, the AI classifier classifies the data into binary classes as malicious and non-malicious data. The non-malicious data is further passed to the next layer, which is the blockchain layer.

### 4.3. Blockchain Layer

A blockchain is a distributed database that preserves an ever-expanding collection of organized entries known as blocks. It is a shared, unchangeable ledger that simplifies the task of documenting transactions and monitoring assets within a network. Here, the blockchain layer offers security and privacy for critical infrastructure data. From the intelligence layer, non-malicious data comes into the blockchain layer, which is stored in the smart contract. The smart contract runs on the solidity compiler v0.8.21 and offers data security by managing the user’s agreement. We employed the Sepolia testnet with MetaMask during the development of the smart contract. The execution of the smart contract to validate the authenticity of IoT-driven critical infrastructure data. We employed various smart contract functions, i.e., addAuthorized(), removeAuthorized(), storeSensorReading(), AuthorizedUsers(), and getSensorReading(). The addAuthorized() function checks the authenticity of the non-attack data. Furthermore, to remove an authorized entity in a smart contract, we used removeAuthorized(). Furthermore, the storeSensorReading() function securely logs sensor data or readings in a decentralized and immutable manner. For the management of approved users or access permissions within the smart contract’s context, we used AuthorizedUsers(). Furthermore, the getSensorReading() function is used for accessing or fetching sensor data or readings stored within the blockchain. Next, the data is passed to the IPFS, which facilitates distributed file storage to track different versions over a time period. We used a file-based web console dashboard, which selects IPFS-content identifiers (CID) from the IPFS bucket and generates the hash value of the deployed smart contract functions. Furthermore, IPFS creates a hash value for the non-malicious data using the secure hash algorithm (SHA)-256 algorithm.

(12) $$\begin{array}{c} \sigma \overset{\mathrm{Smart}\;\mathrm{contract}}{\underset{\mathrm{through}}{\to }}\mathrm{IPFS}\overset{\mathrm{stores}}{\underset{\mathrm{hashvalue}}{\to }}\mathrm{Blockchain} \end{array}$$

Moreover, in the proposed scheme, the IPFS hash is stored as the event log with the associated gas cost, which reduces the mining cost. For example, according to the literature [36], suppose the gas value available is 27,501 units, then the gasCost (wei) is 550,020,000,000,000 wei, which requires gasCost around 0.00055002 ether. Furthermore, the hash value is stored in the blockchain that comes from the IPFS. After that, the data is passed to an application layer, from which an authorized person can access the IoT-based critical infrastructure data.

### 4.4. Application Layer

The layer comprises various critical infrastructures such as water treatment, nuclear power, and thermal plants that use the classified data for further analysis and research. For example, the operators or employees who work in the water treatment plant infrastructure aim to increase the quality of human life by providing pure water. Therefore, they improve the water quality based on the data received from the blockchain. From the blockchain network, they receive non-malicious data that directly affects human life. Nuclear power plants and thermal plants work in a similar manner. Therefore, the proposed architecture is useful in this type of IoT-based critical infrastructure environment.

## 5. Results and Discussion

This section discusses the performance of the proposed architecture by considering different performance parameters, such as accuracy, precision, recall, and F1 score. In addition, this section also displays the experimental setup used while implementing the proposed architecture.

### 5.1. Experimental Setup

In the proposed work, we used the Google Colab application and the Remix integrated development environment (IDE) platform. Google Colab is used to implement and evaluate data for feature selection and classification. First, we have applied the PCA approach with various libraries, such as Matplotlib, Seaborn, Scipy, Python, Pandas, and Numpy, for data preprocessing training, dimensionality reduction, and visualization purposes. Furthermore, we applied XAI to deal with the data poisoning attack on IoT-based critical infrastructure. For the XAI approach, we utilized ELI5, a Python package that provides the weights of the features and allows predictions of scikit-learn linear classifiers and regressors. Lastly, we applied different AI classifiers such as RF, SVM, KNN, perceptron, and GaussianNB using an open-source data analysis library such as scikit-learn for critical infrastructure. Table 2 shows the summary of the hyperparameters for all AI classifiers used in the proposed framework. Moreover, the Remix IDE platform is a tool with a graphical user interface (GUI) that is used for smart contract development. Here, we developed different smart contract functions, such as addAuthorized(), removeAuthorized(), storeSensorReading(), AuthorizedUsers(), and getSensorReading(). Furthermore, we linked smart contract functions with IPFS-based storage.

### 5.2. Feature Selection

**Figure 3:**
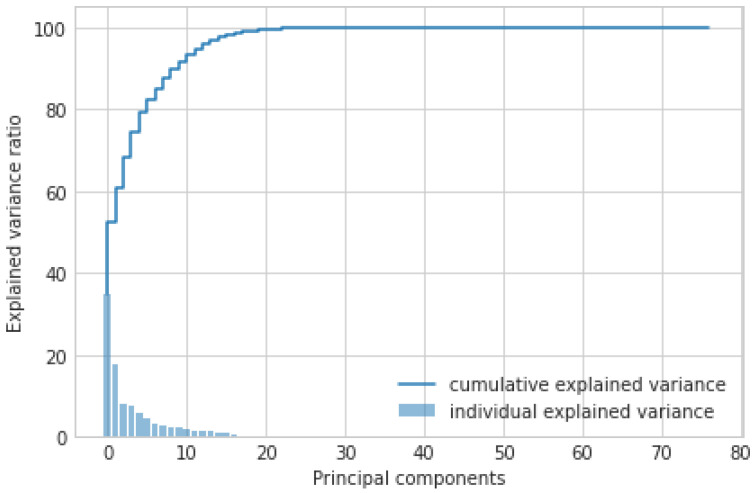
Feature selection using PCA (35 out of 83 features are selected).

**Figure 4:**
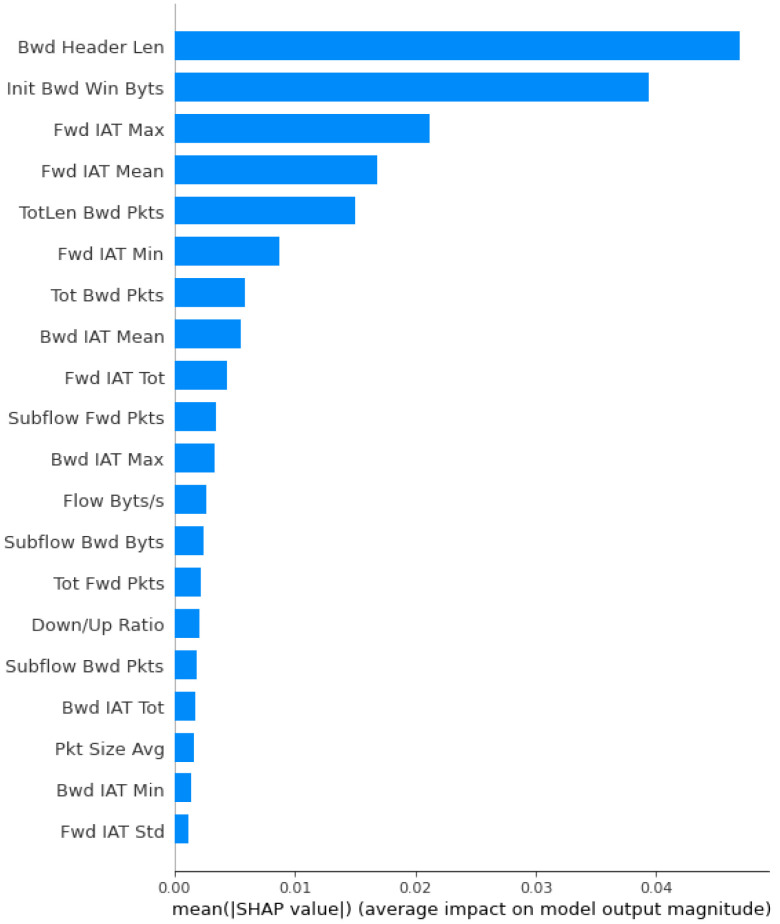
Feature selection using XAI (20 out of 35 features are selected).

- **PCA:** It is an AI approach that transforms the columns of a dataset into a new set of features known as principal components. It effectively compressed the data into fewer feature columns that enable dimensionality reduction. Figure 3 shows the PCA-based feature selection approach. Initially, the dataset contains 83 features used for the IoT-based critical application. However, these large numbers of features make it time-consuming to train our AI model. Therefore, using the PCA approach, we have significantly reduced the number of features from 83 to 35. From the graph, we observed the importance of feature selection to reduce the number of dimensions and improve the performance of the IoT-based critical application.
- **XAI:** Furthermore, we applied XAI to select more specific features based on the feature score. Initially, we identified 35 features out of 83 using the PCA approach. However, we found the requirement to reduce the feature count further to achieve notable results for AI classifiers in terms of accuracy, precision, and F1 score. Figure 4 depicts the feature selection process of the XAI-based approach. From the results, we observed that there are 20 features, such as Bwd header len, Init Bwd win Byts, Fwd IAT Max, Fwd IAT Mean, etc., that are important and have a higher weight than other features. Therefore, we have passed the selected features to the different AI classifiers.

### 5.3. Performance Analysis of AI Classifier

**Figure 5:**
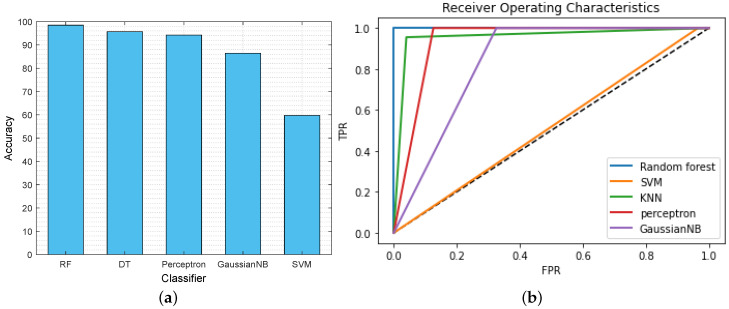
(**a**) Accuracy comparison of AI classifiers. (**b**) ROC of AI classifiers.

This section illustrates the performance of the proposed AI classifier that classifies whether the data is malicious (class 0) or non-malicious (class 1) for the IoT-based critical infrastructure. Figure 5a depicts the comparison of the classification accuracy of the different AI classifiers, such as RF, DT, perceptron, GaussianNB, and SVM. From the graph, we found that, due to the large dataset, SVM is computationally expensive. Moreover, SVM requires more training time, and target classes are overlapped when the dataset has noise. As a result, it becomes slow and ineffective for real-time data classification in IoT-based critical infrastructure. Furthermore, GaussianNB is a probabilistic classifier that applies Bayes’ theorem with strong independence assumptions. This assumption of all features being independent of each other is not the case in the dataset. Therefore, the GaussianNB classifier does not perform well for the given classification problem. In contrast, the selected features are on various scales; RF still performs well and offers higher accuracy than the other classifiers. It provides the probability of belonging to a class of malicious or non-malicious data with 98.46% accuracy. Table 3 displays other parameters, such as precision, recall, and F1 score, to showcase the remarkable performance of the RF classifier.

Furthermore, we have considered the receiver operating characteristics (ROC) curve as a measure of the performance of the AI classifiers. It provides the relationship between sensitivity and specificity after analyzing the classification thresholds. Here, AI classifiers perform better when their ROC curve is nearer the threshold value, i.e., 0.0 (near the y-axis). On the contrary, when the ROC curve is closer to the 45-degree diagonal, the results are not accurate. Figure 5b shows the ROC curve of the different AI classifiers that classify malicious or non-malicious data. The graph concludes that the RF classifier performs well compared with other classifiers because the ROC curve of the RF classifier (blue line) is near the threshold (0.0).

**Figure 6:**
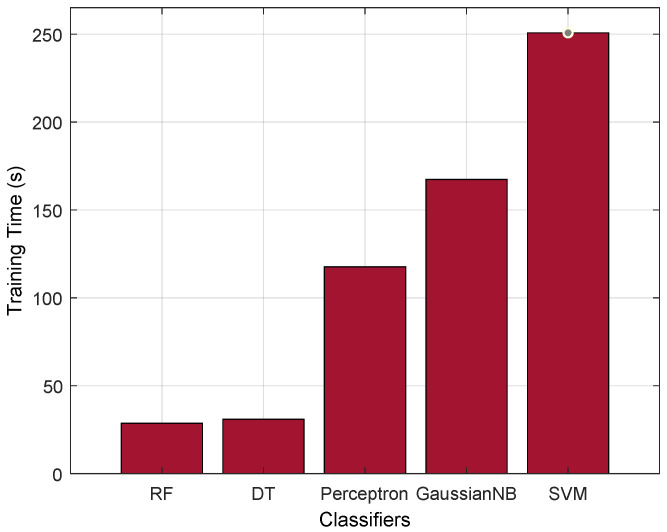
Training time comparison.

Furthermore, to justify the performance of the RF classifier, we evaluate it with the training time parameter. From Figure 6, we can observe that the RF has a better training time, i.e., 28.641 (s), compared with the existing AI classifiers. RF is essentially a collection of multiple DTs, each constructed on a different subset of the training data and with a subset of the available features. The ensemble approach of RF offers advantages such as parallelism and reduction in overfitting, because of which it significantly reduces the AI training time.

**Figure 7:**
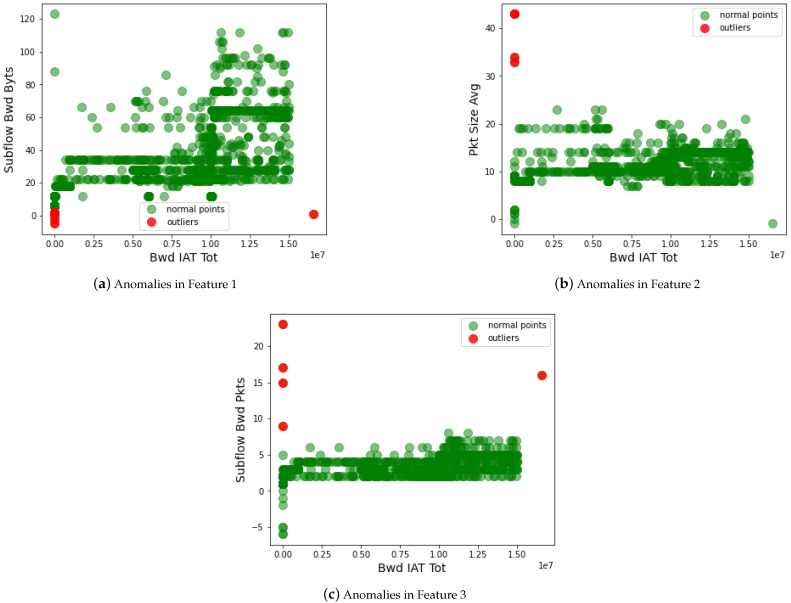
Detecting anomalous data from the feature space (Feature 1-Feature 3) of the dataset.

In addition to statistical analysis, we also measured the security of the AI classifier by removing the anomalous data on the dataset. Formally, this is done before the classification to strengthen the performance of AI classifiers. We analyze the feature space of the dataset using different techniques, such as outlier detection, normalization, distribution of data, and many more. This approach helps to tackle the data poisoning attack on the IoT-based critical infrastructure. Figure 7 shows the performance of the data poisoning attack detection approach. In the dataset, the attacker manipulates the essential reading of IoT data, which deteriorates the regular operation of critical infrastructure. For instance, a criminal (being an attacker) modifies the criminal data (e.g., arrest to not arrest) from the servers of the police department. Such anomalous data needs to be identified and removed from the feature space of the dataset. Therefore, we identify the outlier in the dataset, which helps to improve the security against the data poisoning attack on the dataset. From the graph, we observed that the green dots show actual data of the critical infrastructure, and the red dots are the outliers in the dataset. The outlier manipulates the results of the actual information of the dataset. Due to this, the AI classifier misleads and performs the wrong classification. Such outliers are removed from the dataset, and only the correct data is forwarded to the AI classifiers for data training.

Table 4 displays the accuracy comparison between the existing state-of-the-art approaches and the proposed work. In [2], the authors used an SVM classifier to detect attack and non-attack data in IoT-enabled critical infrastructure. They are getting 97.7% on their classification task. Similarly, the authors of [23] utilized a deep learning technique, i.e., a convolutional neural network (CNN), for the intrusion detection system in the water treatment plant. Their findings show that their approach achieves an accuracy of 96.51% compared with other baseline works. Alternatively, in [28,37,38], the authors are achieving noteworthy accuracy in bifurcating malicious and non-malicious critical infrastructure data. The proposed work employs XAI techniques for efficient feature selection and anomaly detection to remove anomalies from the utilized dataset. It is applied prior to the AI training, and because of these add-ons, the proposed work has better performance compared with the existing state-of-the-art works. Consequently, the proposed work achieves 98.46% accuracy compared with the existing baseline works, as mentioned in Table 4.

### 5.4. Performance Analysis of the Blockchain

**Figure 8:**
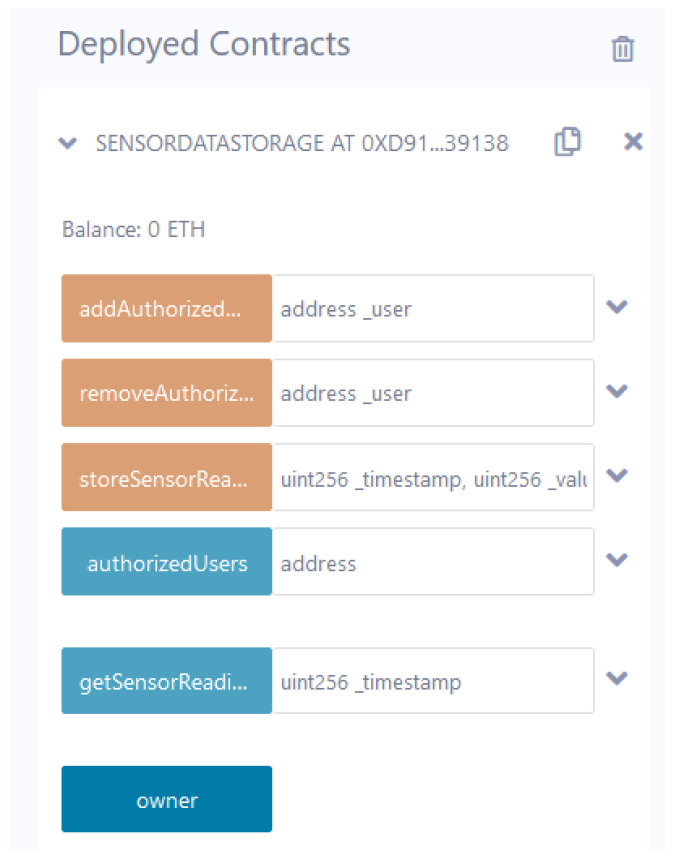
Deployed smart contract.

This section analyzes the performance of the blockchain network in terms of scalability. The designed smart contract is integrated with the IPFS (on-site storage) via an open-source platform-Filebase. It offers a Filebase bucket where a content identifier (CID) is generated for each file. The CID is further integrated with the Remix development environment. Furthermore, the smart contract is compiled using a solidity compiler and deployed on a Sepolia test network. Figure 8 shows the deployed smart contract comprised of authorization entities and storing the sensitive data of critical infrastructure on the Sepolia test network.

**Figure 9:**
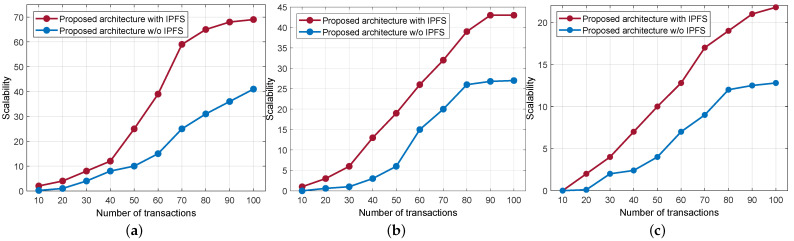
Scalability comparison with IPFS and without IPFS. (**a**) CPU at 5% utilization. (**b**) CPU at 50% utilization. (**c**) CPU at 80% utilization.

Since the smart contract is attached to the IPFS network, all sensitive data gets stored in its secure storage, and the associated hash of the original data is forwarded to the blockchain’s immutable ledger. As a result, it improves the response time of the blockchain network. Since the hashed data size is comparably smaller than the original data size, it makes for efficient storage and retrieval. Based on the response time, we computed the scalability of the blockchain network by varying the CPU utilization. As a rule of thumb, the lower the response time, the higher the scalability. In that view, Figure 9 shows the scalability of the blockchain network. We compared scalability with the IPFS-enabled blockchain and without IPFS by varying CPU utilization, i.e., at 5%, 50%, and 80%. From the figure, it is evident that the IPFS-based blockchain offers better scalability compared with the conventional blockchain.

## 6. Conclusions

In this paper, we propose an AI and blockchain-driven intelligence anomaly detection architecture that provides security in the IoT-based critical infrastructure. Towards this aim, a standard dataset is utilized, which comprises data exchange between different IoT-based critical infrastructures, such as water treatment plants, nuclear power plants, and thermal plants. Furthermore, the dataset is pre-processed using diverse data pre-processing techniques that help enhance the overall performance of the proposed architecture. From the detailed analysis, we identified that there is a need to apply future selection methods to reduce the computation overhead caused by less important features and bulky datasets. Thus, we applied feature section techniques such as PCA and XAI that perform dimensionality reduction on the pre-processed dataset. Furthermore, we detect data poisoning attacks in the AI models that deteriorate the performance of AI training. As a result, AI classifiers such as RF, DT, SVM, perceptron, and GaussianNB classify the inaccurate results. To tackle this issue, we proposed anomaly detection that identifies the anomalous data from the feature space of the dataset and offers accurate results. The results show that the RF classifier achieves 98.46% accuracy compared with the other AI classifiers. Moreover, to enhance the security of the classified data, we used the IPFS-driven blockchain network, which offers secure storage of the critical infrastructure’s data. 

In future work, we will improvise the security implications in the IoT-based critical infrastructure by utilizing synthetic datasets, which will simulate in a Matlab environment. Furthermore, we will make a comparative analysis of our synthetic dataset results with the standard real-time results.

## Figures and Tables

**Table 1:** A comparative analysis of the proposed work with the existing state-of-the-art works.

Author	Year	Objective	Methodology	1	2	3	4	5	Pros	Cons
Chang et al. [17]	2019	Anomaly detection in ICS	K-means and convolutional autoencoder	*X*	*X*	*X*	*X*	*X*	The adopted approaches gives higher accuracy on a gas pipeline and water storage tank dataset	PCA and XAI has not considered
Alhaidari et al. [20]	2019	Secure supervisory control and data acquisition systems against DDoS attack	NB, RF, and J48	*X*	*X*	✔	*X*	*X*	Among all three approach RF gives higher accuracy	They have focused on the feature selection
Elnour et al. [23]	2020	Hybrid attack detection scheme for water treatment plant	Isolation Forest and CNN	*X*	*X*	✔	*X*	*X*	The scheme detects maximum attacks, reduces the computational complexity and increases the accuracy compared with the other approaches	Not discussed about data poisoning attack on the dataset
Rakesh et al. [24]	2021	Monitor water quality using ML approach	NB, RF, and LR	*X*	*X*	*X*	*X*	*X*	The scheme compares the simulation results with the experimental results	Not considered feature selection
Khan et al. [28]	2021	Facilitate resource-efficient solution to the IoT application	ANN, SVM, DT, and DELM	*X*	*X*	*X*	✔	*X*	The scheme offer security and protection to the smart home	Not considered data positioning attack and IPFS-based storage
Puthal et al. [25]	2022	User-centric security and fake data identification for IoT-based critical infrastructure	DT	*X*	*X*	*X*	*X*	*X*	They proposed theoretical and experimental solution that resist brute force, DDoS, and replay attack	Not compared the DT results with other AI approaches such as SVM, RF, XGBoost, etc.
Narayanan et al. [26]	2022	Anomalies detection in smart cyber-physical systems	Automatic behavioural abstraction technique based on neural networks	*X*	*X*	✔	*X*	*X*	The scheme detects the maximum number of attack with 1 percent a false positive rate	They have not taken feature selection approaches and data poisoning attack
Ragab et al. [31]	2022	To secure the industrial control system	SVM, RF, Adaboost, KNN, and BDLE-CAD	*X*	*X*	*X*	✔	*X*	They applied chimp optimization based feature selection that increases the accuracy	Not considered data poisoning attack for dataset
Gu et al. [29]	2023	Quality control in manufacturing process	KNN, XGboost, ANN, XGB Max	*X*	*X*	*X*	✔	*X*	The proposed scheme prevents DoS, man in the top, DDoS, and brute force.	Not considered data poisoning attack.
The proposed architecture	2023	Secure data dissemination architecture	RF, DT, SVM, perceptron, and GaussianNB classifier	✔	✔	✔	✔	✔	Accurate, efficient, secure, and reliable architecture for IoT-based critical infrastructure	-

Parameters- 1: PCA, 2: XAI, 3: Anomaly detection, 4: Conventional blockchain, 5: IPFS-based blockchain.

**Table 2:** Summary of hyperparameters used by AI classifiers.

AI Classifiers	Parameters Used
RF	n_estimators: 200, max_depth: 5
DT	criterion: [‘gini’], splitter: [‘best’, ‘random’]
SVM	gamma: [‘auto’], probability: True, kernel: [‘rbf’]
Perceptron	alphafloat: [0.0001], l1_ratiofloat: [0.15]
GaussianNB	priors: None, var_smoothing: 1 $\times 10^{-9}$

**Table 3:** Comparison of AI models performance for different metrics.

AI Models	Accuracy (%)	Precision (%)	Recall (%)	F1 Score (%)
**RF**	98.46	97.56	95.55	96.65
**SVM**	59.76	59.41	57.65	58.89
**Decision tree**	95.71	97.56	96.53	94.45
**Perceptron**	94.22	92.23	87.23	93.32
**GaussianNB**	86.42	81.23	78.34	85.43

**Table 4:** Accuracy comparison with the existing state-of-the-art works.

Authors	[2]	[23]	[28]	[37]	[38]	Proposed
Accuracy	97.7%	96.51%	92.01%	92.3%	95.6%	98.46%

## Data Availability

No data is associated with this research work.
